# Coalescence Processes of Droplets and Liquid Marbles

**DOI:** 10.3390/mi8110336

**Published:** 2017-11-20

**Authors:** Jing Jin, Chin Hong Ooi, Dzung Viet Dao, Nam-Trung Nguyen

**Affiliations:** Queensland Micro- and Nanotechnology Centre, Nathan Campus, Griffith University, 170 Kessels Road, Brisbane, QLD 4111, Australia; jing.jin3@griffithuni.edu.au (J.J.); chinhong.ooi@griffithuni.edu.au (C.H.O.); d.dao@griffith.edu.au (D.V.D.)

**Keywords:** droplet, liquid marble, coalescence, digital microfluidics, microreactors

## Abstract

The coalescence process of droplets and, more recently, of liquid marbles, has become one of the most essential manipulation schemes in digital microfluidics. This process is indispensable for realising microfluidic functions such as mixing and reactions at microscale. This paper reviews previous studies on droplet coalescence, paying particular attention to the coalescence of liquid marbles. Four coalescence systems have been reviewed, namely, the coalescence of two droplets freely suspended in a fluid; the coalescence of two sessile droplets on a solid substrate; the coalescence of a falling droplet and a sessile droplet on a solid substrate; and liquid marble coalescence. The review is presented according to the dynamic behaviors, physical mechanisms and experimental parameters of the coalescence process. It also provides a systematic overview of how the coalescence process of droplets and liquid marbles could be induced and manipulated using external energy. In addition, the practical applications of liquid marble coalescence as a novel microreactor are highlighted. Finally, future perspectives on the investigation of the coalescence process of liquid marbles are proposed. This review aims to facilitate better understanding of the coalescence of droplets and of liquid marbles as well as to shed new insight on future studies.

## 1. Introduction

Digital microfluidics (DMF) is an emerging liquid-handling technology dealing with the manipulation of small discrete droplets [1]. In DMF, microliter-sized droplets serve as miniaturized reaction chambers. This process has numerous advantages such as minimum reagent requirement, fast response rates, low cross-contamination and, more importantly, the capability of performing parallel tests. These advantages make DMF a perfect candidate for practical lab-on-a-chip (LOC), micro total analysis systems (μTAS) and point-of-care (POC) diagnostic devices in clinical use [2,3,4,5,6,7]. To date, DMF can be classified, based on its droplet types, into droplet-based DMF and liquid marble-based DMF [3].

Droplet-based DMF generally involves tasks such as dispensing droplets, moving droplets, merging droplets or mixing contents within a droplet. Among these tasks, droplet merging, i.e., droplet coalescence, is the most common technique and has been studied both qualitatively and quantitatively. Droplet coalescence is the process where two or more droplets merge during contact to form a single droplet. This extreme case of binary droplet collision involves large deformations and the rupture of the interface separating the droplets [8]. The coalescence or fusion process of droplets has drawn extensive attention from many researchers as it is of fundamental importance for understanding raindrop formation [9], sintering in metallurgy [10], emulsification [11], ink-jet printing, spray coating, combustion of fuel sprays, waste treatment [12,13,14,15] and more recently, microfluidics [16]. In terms of its practical use in DMF, droplet coalescence is an essential procedure that enables desired droplet-based functions such as micromixing [17,18,19,20] and microreaction [4,21,22] to be achieved in LOC devices for chemical and biological assays.

**Figure 1:**
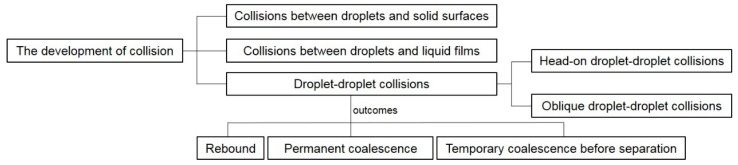
The development of droplet collisions.

To understand the dynamic behaviors and physical mechanisms of droplet coalescence, a wide range of experimental and theoretical work focusing on impacts between droplets and solid surfaces, liquid films or other droplets has been conducted. The earliest study can date back to the late 19th century, with the focus on droplets impacting on to flat plates and liquid surfaces [23,24,25]. Since then, investigations have been expanded to include the impacts between droplets and solid surfaces [26,27,28], collisions between droplets and liquid films [29,30,31,32,33] and head-on or oblique droplet–droplet collisions [34,35,36,37,38] (Figure 1). In particular, the coalescence of droplets has been actively studied due to the emergence of new research ideas and methodologies in the last decade. These include droplet coalescence induced by surface tension [39] or by surface diffusion [40], and merging of droplets in microfluidic channels [41,42,43]. The few existing review papers on droplet coalescence have mainly emphasized droplet impact or collision regimes [37,44,45,46] and droplet coalescence in microfluidic systems [47,48,49], and do not provide comprehensive coverage of the extensive work conducted in this area.

Another growing field in DMF is the use of a liquid marble (LM) as a discrete platform. A LM is a small-volume liquid droplet coated with one or multiple layers of micro- or nanometer-sized powders. Most coating powders are hydrophobic, which allows the LM to be manipulated like a soft solid [50,51,52,53,54,55]. Due to its non-wetting property, a LM exhibits very low friction with its carrier surface. This feature allows LMs to move easily with zero loss across solid and even liquid surfaces.

A LM can be formed simply by rolling a liquid droplet over a bed of hydrophobic powder. The powder assembles itself on the droplet, effectively creating a protective coating. LMs can rest steadily on a solid substrate or even float on a liquid surface for some time before dissipating via evaporation [56,57,58]. As a discrete droplet separated from the surrounding environment, a LM can be actuated by external forces [59]. In particular, a LM with a magnetic coating or consisting of magnetic fluid can be opened and closed reversibly in a magnetic field, which enables it to function as a novel microreactor [60]. For DMF, samples within LMs can be transported with minimal loss and practically zero contamination, which is a major advantage compared to bare droplets. A detailed description of LMs as a novel digital microfluidic platform for chemical and biological assays will be discussed in Section 4. Although numerous technical and review papers on the fabrication [61], manipulation [55] and properties [51] of LMs have been published, no review exists on LM coalescence. The present paper thus attempts to provide a comprehensive understanding of droplet coalescence, with particular attention paid to LM coalescence.

The review is organised as follows. Section 2 discusses fundamental physics of droplet coalescence and LM coalescence based on four coalescence systems. Section 3 lists some coalescence examples in industrial applications. Section 4 mainly reviews the diverse applications of LM coalescence for chemical and biological assays as a novel digital microfluidic platform. Section 5 concludes the review and proposes future perspectives in the investigation of LM coalescence.

## 2. Fundamental Physics of the Coalescence Process of Droplets and Liquid Marbles

**Figure 2:**
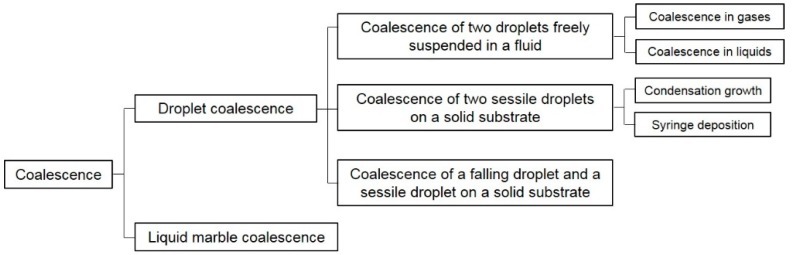
The coalescence systems reviewed in this paper.

Based on previous studies on the coalescence process of droplets and LMs, the process can be categorised into four different systems: (i) Coalescence of two droplets freely suspended in a fluid; (ii) Coalescence of two sessile droplets on a solid substrate by condensation growth or syringe deposition; (iii) Coalescence of a falling droplet and a sessile droplet on a solid substrate; and (iv) LM coalescence (Figure 2). Some terms used in this review are defined as follows. First, the dimensionless Weber number (*We*) is defined as:

(1) $$We=\frac{\rho v^{2}D}{\sigma },$$

where *ρ* and *σ* are the density and surface tension of the droplet fluid respectively; *v* is the relative droplet velocity; and *D* is the droplet diameter. The Weber number is regarded as the relative ratio of the inertia of the droplet fluid to the corresponding surface tension, which is often used to characterize droplet coalescence in microfluidics. Second, another important dimensionless parameter is the Reynolds number (*Re*) that is defined as:

(2) $$Re=\frac{\rho vD}{\mu },$$

where *μ* is the dynamic viscosity of the droplet fluid. It is the ratio of the inertial forces to viscous forces within the droplet fluid. When *Re* < 1, the droplet coalescence dynamics is dominated by viscous forces. While for *Re* > 1, inertial coalescence occurs. Third, the Ohnesorge number (*Oh*) is a dimensionless number that relates the viscous forces to inertial and surface tension forces:

(3) $$Oh=\frac{\mu }{\sqrt{\rho \sigma D}}=\frac{\sqrt{We}}{Re},$$

### 2.1. Mechanisms of Droplet Coalescence

#### 2.1.1. Coalescence of Two Droplets Freely Suspended in a Fluid

The majority of research on coalescence of free-suspended droplets has been performed on unconfined droplet collisions in air, water or other fluids. In these experiments and simulations, the droplets were not in contact with a solid substrate. Therefore, the complexity introduced by the presence of a three-phase contact line has not been considered. This is the ideal model for understanding the dynamics and mechanisms of droplet coalescence and provides inspiring ideas to further research on droplet coalescence on a solid substrate.

Ashgriz and Poo [62], Jiang et al. [63] and Qian and Law [64] conducted various experiments of binary droplet collision in different gases to investigate the dynamics of free-suspended droplets coalescing in air. Three collision outcomes were identified over a wide range of Weber numbers, off-center distances and other parameters, namely (i) rebound, (ii) permanent coalescence and (iii) temporary coalescence before separation. Nobari et al. [8] simulated the head-on collision of equal-sized droplets using a front tracking/finite difference technique. The authors focused on the transition boundary between coalescence and rebound. Furthermore, Mashayek et al. [65] considered coalescence through the collision of two liquid drops by using a Galerkin finite element method in conjunction with the spine-flux method proposed in their previous study.

As a complement to the above studies, Willis and Orme [66,67] investigated the collision of viscous binary droplets in a vacuum environment to understand the dynamics related to aerodynamic and viscous effects. Their results showed that the critical Weber number, which distinguished permanent coalescence with coalescence followed by separation, was several orders of magnitude higher than that of experiments conducted in a standard ambient environment with lower-viscosity liquids.

**Figure 3:**
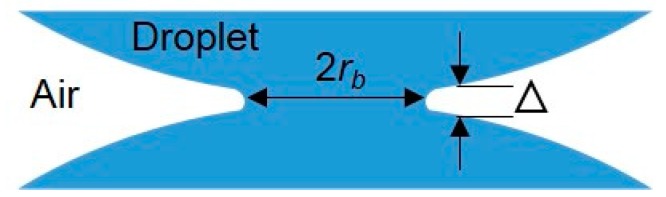
The close-up of the small bridge (or neck) connecting two droplets. 2 *r_b_* is the diameter of the bridge along the initial contact line and ∆ is the bridge width.

**Figure 4:**
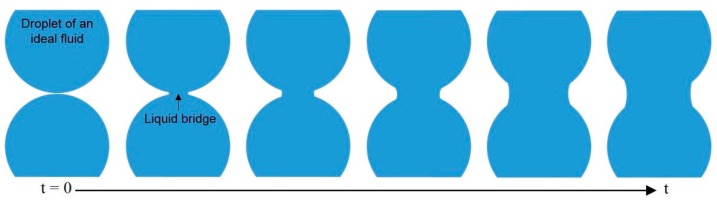
Schematic of the time evolution of the liquid bridge connecting two droplets.

There were some inspiring numerical studies on droplet coalescence driven by surface tension published around the end of 20th century. As the merging motion of two drops was always viscously dominated in the early stage, Eggers et al. [68] theoretically explored the early-time behavior of the radius *r_b_* of the small bridge connecting the two drops (Figure 3). They found that the flow inside the drops was driven by a highly curved meniscus with the length of 2π*r_b_* and the width of ∆ ≪ *r_b_* around the bridge, from which they concluded that the behavior of three-dimensional droplet coalescence was asymptotically equivalent to its two-dimensional counterpart. They also studied in numerical detail the more general case of the coalescence of droplets within an external viscous fluid. Furthermore, Duchemin et al. [69] simulated the coalescence process of two droplets of an ideal fluid driven by surface tension. They argued that the minimum radius *r_b_* of the liquid bridge connecting two droplets was proportional to the square root of time *t* by averaging a number of discrete experiments (Figure 4). This is qualitatively similar to the numerical result proposed by Oguz and Prosperetti [70], who simulated the coalescence of two flat inviscid liquid surfaces connected at a neck region and observed the entrapment of bubble rings.

**Figure 5:**
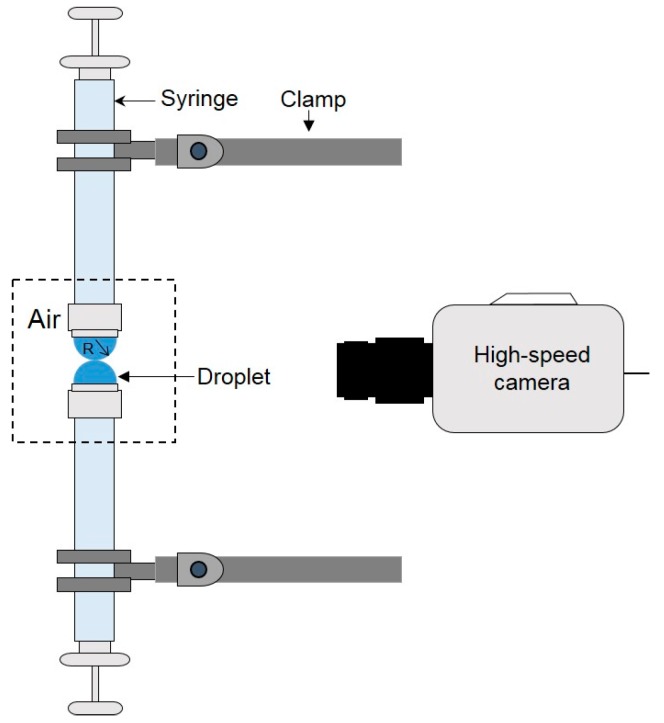
Schematic of the droplet coalescence experiment in air.

To understand the dynamics of liquid droplet coalescence in microfluidic devices driven by surface tension, Wu et al. [16] studied the early-time evolution of the liquid bridge formed upon the initial contact of two liquid drops in air during coalescence (Figure 5). Among three commonly used fluids, deionized (DI) water, water–glycerol mixture and methanol, they found in all instances that the liquid bridge radius *r_b_* followed the scaling law of *r_b_* ∝ *t*^1/2^ in the inertial regime, which is consistent with Eggers et al’s finding [68]. Their further experiments demonstrated that such a scaling law was robust regardless of the viscosity and surface tension of the fluids. However, these fluid properties did change the shape of the interface between the air and the drops near the liquid bridge. In addition, the coefficient of the scaling law, *r_b_*/*t*^1/2^, fell into the range of 1.03–1.29, which is lower than that predicted by the numerical simulation of Duchemin et al. [69] for inviscid drop coalescence. Similarly, Thoroddsen et al. [71] used an ultra-high-speed video camera to study the coalescence of a pendent and a sessile drop on vertically aligned metal tubes, over a range of droplet sizes and liquid viscosities.

Aarts et al. [72] quantitatively investigated the coalescence of two droplets as well as a droplet with its bulk fluid phase in two different systems. One of them is a molecular system with a variable viscosity and the other is a colloid–polymer mixture with an ultralow surface tension. At large viscosities or small surface tensions, the authors observed that the opening of the liquid bridge initially proceeded at a constant speed set by the capillary velocity *σ*/*μ*, where *σ* and *μ* are the surface tension and dynamic viscosity of the fluid respectively. In particular, the team succeeded in observing purely viscous coalescence, where the radius *r_b_* of the liquid bridge grew linearly with time *t*, as opposed to inertial coalescence for which *r_b_* ∝ *t*^1/2^. Interestingly, for the fluid with viscosity of 50 mPa∙s, the crossover between the viscous and inertial regimes could be observed. Aarts et al. concluded that the coalescence dynamics of droplets was driven by surface tension, and slowed down by viscosity for low Reynolds numbers and by inertia for high Reynolds numbers.

Due to the rapid motion of the liquid inside droplets and the awkward view angle during coalescence, it is difficult to characterize visually the dynamics of the initial stage of droplet coalescence in experiments. Consequently, Case and Nagel [73] employed an electrical method to study the fluid bridge between two merging droplets with low viscosity at much shorter times of *τ*~10 ns, which is better than the shortest times accessible by previous imaging studies. After the measurement of growth dynamics of the bridge, they observed a new asymptotic regime that was inconsistent with previous theoretical predictions. Furthermore, Case [74] used the same electrical method to study the early stages of coalescence of two saltwater drops in air by measuring the resistance and capacitance of the system during this coalescence event. Paulsen et al. [75] also used a similar electrical method and high-speed imaging to probe droplet coalescence down to 10 ns after two droplets contacted each other. By varying the liquid viscosity over two magnitudes, they concluded that, at a sufficiently low approach velocity, the droplets coalesced with an unexpectedly late crossover time between a regime dominated by viscous effects and another dominated by inertial effects.

The corresponding numerical work done by Sprittles and Shikhmurzaev [76] simulated the process of coalescence of two identical liquid droplets in the framework of two different mathematical models. One is the conventional model involving the formation of a liquid bridge connecting the two drops and the subsequent evolution of the single body of fluid driven by capillary forces. The other is the interface formation/disappearance model. The authors showed that the recently reported electrical measurements probing the very early stages of the process [75,77] were better described by the latter model. Based on the numerical results, Sprittles and Shikhmurzaev suggested new theory-guided experiments that would help to further elucidate the details of the coalescence phenomenon.

**Figure 6:**
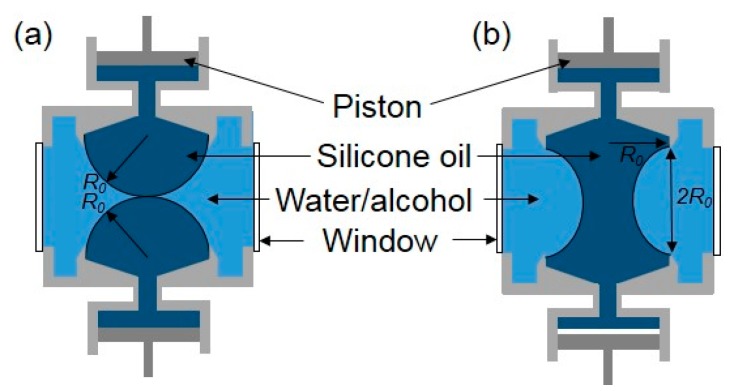
The Plateau tank for the droplet coalescence experiment in silicone oil. (**a**) The configuration of the droplets touching each other; (**b**) the equilibrium shape of the droplets after coalescence.

As for droplet coalescence in liquids, Yao et al. [14] reported the early stage of coalescence of two highly viscous silicon oil droplets immersed in a water–alcohol mixture of the same density to eliminate the effect of gravity. The viscosity of droplets was sufficiently large that measurements on the radius of the neck between the drops could be made under the conditions of Stokes flow. In their experiments, attention was restricted to the consideration of two drops with the same radius *R*_0_ approaching each other with a very small relative velocity (Figure 6).

**Figure 7:**
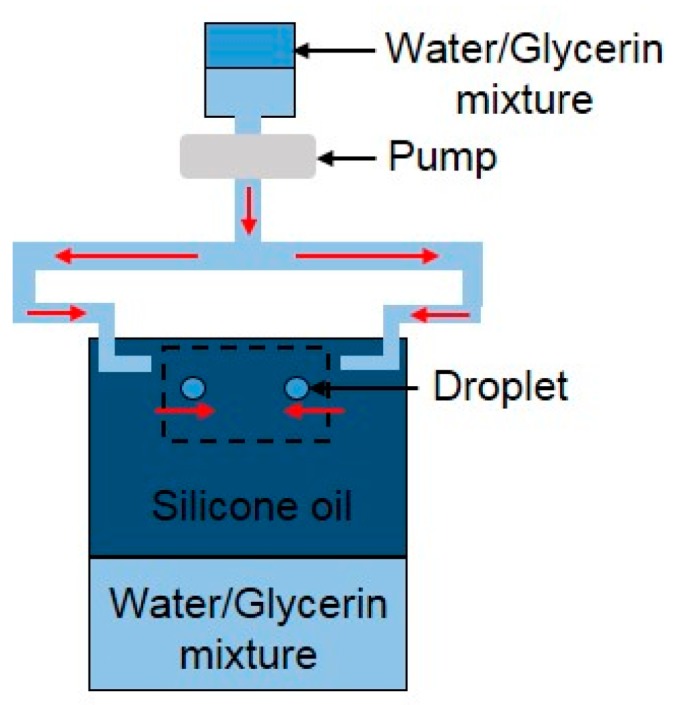
Schematic of flow facility for binary droplet collision in water–glycerin mixture.

Similarly, Kim and Longmire [78] conducted experiments on binary droplet collisions within an index-matched liquid. The water/glycerin mixture droplet pairs were injected horizontally into silicone oil and travelled downward before colliding under the effect of gravity (Figure 7). The team employed a dual-field high-speed particle image velocimetry measurement system to quantify droplet trajectories and overall collision conditions while examining detailed velocity fields at the collision interface simultaneously. The coalescence was found to result from a combination of vortical flow within drops and strong drop deformation with the characteristic of higher Weber numbers. The flow through the centers of opposing ring vortices, which were strengthened by drop deformation, enhanced drainage of the thin film in the impact region, leading to film rupture and subsequent droplet coalescence.

In terms of droplet coalescence in a fluid flow, Leal’s research group [49,79,80] summarized experimental studies of flow-induced coalescence of viscous drops in a viscous fluid when inertial effects were absent. These studies were based on visual observations of small drops that collide in a linear flow generated by a four-roll mill, which ensured the bulk-phase rheological properties of fluids are Newtonian. Under these conditions, droplet pairs coalesced when collisions were gentle: the drops did not deform significantly, and coalescence occurred at the point of contact. They also simulated the coalescence of two equal-sized deformable droplets in an axisymmetric flow by a boundary integral method. Bremond et al. [41] investigated the destabilization process of an emulsion under flow in a microfluidic device. They demonstrated an unusual phenomenon when the two droplets collide, namely coalescence occurred during the separation phase and not during impact. Scarbolo et al. [42] also conducted similar research to investigate the coalescence and breakup of large deformable droplets dispersed in a wall-bounded turbulent flow. Depending on the different values of *We*, the authors observed two different regimes: when *We* < 1 in the simulations, coalescence dominated until droplet–droplet interactions were prevented by geometric separation; when *We* > 1, there was a permanent dynamic equilibrium between coalescence and breakup events.

Generally, merging droplets in a fluid tend to keep near spherical shapes just before coalescence occurs. After the contact, a liquid bridge is formed between the two coalescing droplets. This meniscus bridge grows rapidly and then slowly relaxes to an equilibrium shape, which follows different scaling laws for inertial coalescence and viscous coalescence, until the formation of a bigger daughter droplet. The approaching velocity of the droplets is usually taken to be zero and the dynamic effect of air can be neglected. However, the outer liquid does affect the coalescence process in flows. The high-speed camera has been adapted extensively to record the droplet coalescence process. Novel detection methods, such as the electrical method, were recently developed for studying the dynamics of the initial stage of droplet coalescence. The research on the coalescence of droplets freely suspended in a fluid also facilitates the study of droplet coalescence involving a solid substrate.

#### 2.1.2. Coalescence of Two Sessile Droplets on a Solid Substrate

In many other cases, however, the coalescing droplets were not freely suspended in air or liquids, but were in contact with a solid substrate. The challenge of understanding the fluid dynamics of droplet coalescence on a substrate lies in the complexity introduced by the presence of the substrate. First, the geometry of the sessile drop is no longer a sphere with an axisymmetric bridge, but a spherical cap with a certain contact angle *θ*. Second, the substrate slows down the liquid transport towards the bridge [81] and results in the motion of a contact line [46]. It is still not clear whether this contact line motion affects the initial stages of coalescence, and different predictions for the contact angle *θ* dependence have been reported [82,83]. Ristenpart et al. [81] argued that the main flow direction was parallel to the substrate and oriented towards the bridge based on numerical simulations, which simplifies the description of droplet coalescence but still remains to be validated experimentally.

**Figure 8:**
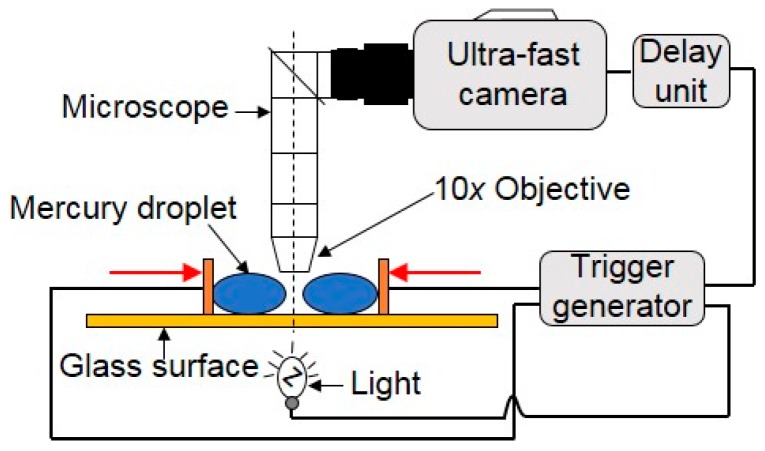
The ultra-fast analog camera set-up used to obtain a magnified view of the contact region of two mercury droplets.

Menchaca-Rocha et al. [39] observed the coalescence of two small near-spherical mercury droplets with very low velocity by placing them on an optimized rough, horizontal glass surface (Figure 8). It was found that the surface-shape evolution for the coalescence of mercury droplets was driven solely by surface tension. The neck-size evolution was observed to follow a scaling law with an exponent ranging from 0.41 to 0.55, which is close to the previous finding by Eggers et al. [68]. Menchaca-Rocha et al. further made both qualitative and quantitative comparisons between the time evolution of the overall-surface shape and computations of the Navier–Stokes equation with a free surface. They found that the experiment data agreed well with the simulation.

**Figure 9:**
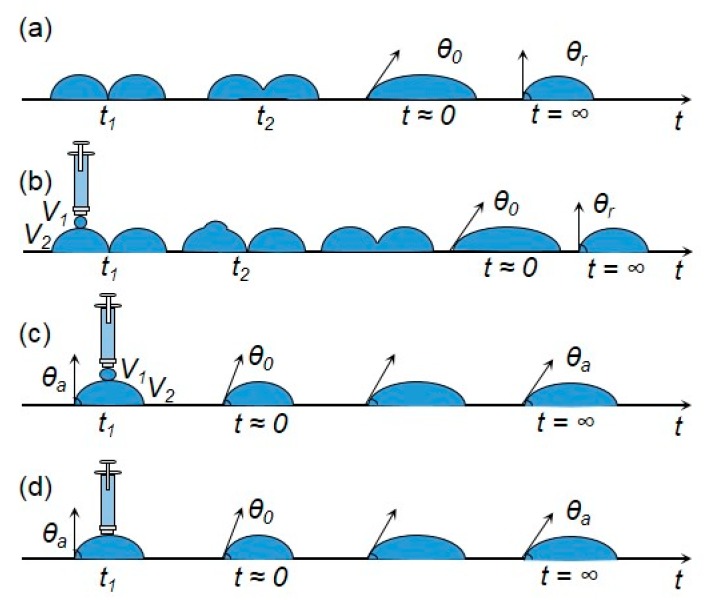
Sketch of (**a**) coalescence process in condensation growth experiment; (**b**) coalescence process in syringe-deposition experiment; and (**c**,**d**) spreading of droplet in syringe-deposition experiment.

Andrieu et al. [84] experimentally and theoretically studied the kinetics of two water droplets coalescing on a flat solid substrate under partial-wetting conditions. These drops were in a chamber full of nitrogen saturated with water. The droplets grew by condensation and eventually touched each other and coalesced. In this experiment, a new elongated composite drop was rapidly formed and then exponentially relaxed to an equilibrium hemispherical cap. Moreover, the characteristic relaxation time, which was proportional to the drop radius at final equilibrium, appeared to be nearly 10^7^ times larger than the bulk capillary relaxation time. The authors explained this phenomenon using a model that involved an Arrhenius kinetic factor resulting from a liquid–vapor phase change in the vicinity of the contact line. Narhe et al. [85] also compared the dynamics of coalescence of two water sessile droplets with the spreading dynamics of a single drop in a partially wetting regime. In their experiment, the coalescence of two drops was studied either in (i) a condensation chamber, where droplets grew by condensation and coalesce when they contacted each other; or (ii) by adding a small drop on top of one of two neighboring drops by a microsyringe (Figure 9).

Narhe et al. found that the relaxation time depended a lot on the drop size, initial conditions and surface properties (such as contact angle and roughness), which could reach several tenths of seconds. It is noteworthy that the relaxation dynamics was slower by 5–6 orders of magnitude than the bulk hydrodynamics predicted, which was attributed to the high dissipation in the contact line vicinity. The research team also did other experimental and theoretical research on the coalescence of sessile droplets [86,87] and distinguished the three stages of the droplet coalescence as follows: (i) an initial stage, where the contact line did not move appreciably. A liquid bridge (or neck) nucleated between the parent drops and grew linearly with time *t* perpendicular to the substrate and with *t*^1/2^ parallel to the substrate; (ii) an intermediate stage where the contact line started to move and where the bridge relaxed exponentially, eventually creating an ellipsis-like drop; (iii) a final stage where, limited by the contact line motion, the drop slowly relaxed to a circular shape.

**Figure 10:**
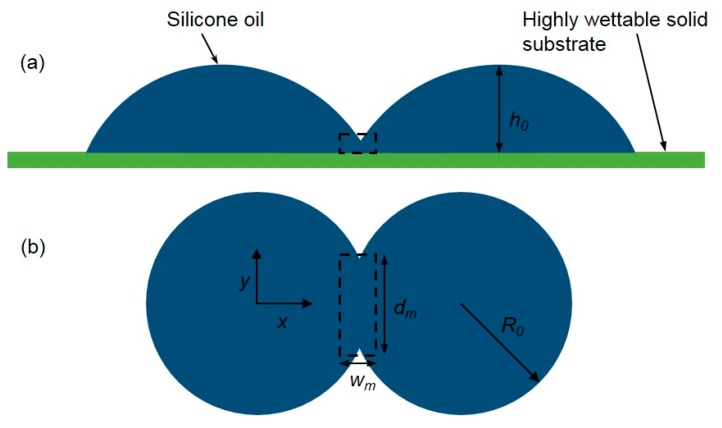
Sketch of two silicone oil droplets coalescing on a highly wettable flat substrate. (**a**) Elevation view; (**b**) plan view. Dashed lines indicate the control volume around the meniscus bridge.

Ristenpart et al. [81] investigated the early-time coalescence dynamics of two spreading viscous silicone oil droplets on a highly wettable flat substrate (Figure 10). Upon contact, surface tension drove a rapid motion perpendicular to the line of centers that joins the droplets and decreased the total surface area. It was found that the width of the growing meniscus bridge between these two droplets exhibited power-law behavior, growing at early times as *t*^1/2^, which supports the finding of previous research [14,16,68,69]. Moreover, the growth rate heavily relied on both the radii and heights of the droplets at contact, scaling as *h*_0_^3/2^/*R*_0_, which differs significantly from the behavior of freely suspended droplets. Hernandez-Sanchez et al. [83] studied the coalescence of viscous silicon oil droplets on a horizontal microscope glass slide both experimentally and theoretically. Besides, Sui et al. [88] considered the growth rate of the height of the connecting bridge in rapid surface-tension-driven coalescence of two identical droplets attached on a partially wetted substrate.

**Figure 11:**
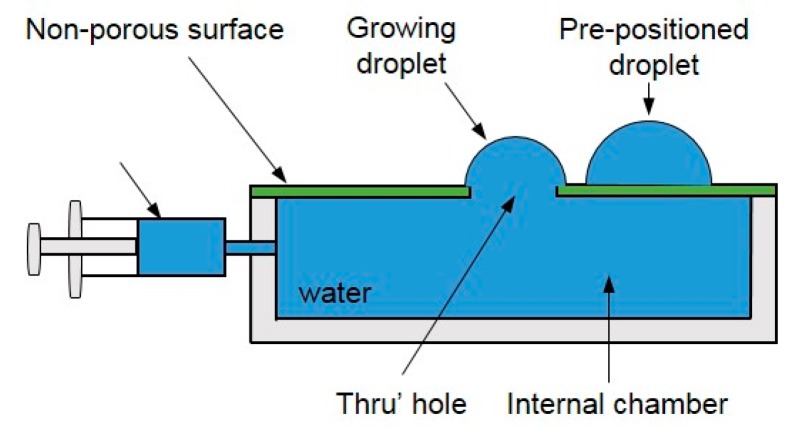
Schematic of the droplet coalescence apparatus using syringe injection.

Kapur and Gaskell [13] investigated experimentally the coalescence of a pair of partially wetting droplets on a non-porous surface (Figure 11). These droplets merged and evolved to a final state with a footprint that is peanut like in shape, with bulges along the longer sides resulting from the effects of inertia during the spreading process. In the early stage of the coalescence, a traveling wave propagated from the point of initial contact up the side of each droplet as liquid was drawn into the neck region, and only when it reached the apex of each droplet, did their heights start to decrease. Lee et al. [82] paid attention to the coalescence experiments of two tiny droplets on partially wettable substrates. The processes were entirely dominated by viscous forces. There are also some studies on the mixing of fluids inside droplets. Lai et al. [89] investigated the fluid dynamics of head-on collisions between a moving droplet and a stationary droplet on a planar surface with a wettability gradient. The authors found the mixing of fluids was passively achieved through convective mass transfer caused by the release of surface energy during coalescence, and diffusive mass transfer as well. Similar work has also been done by Nilsson et al. [90], who focused their study on the effect of contact angle hysteresis on droplet coalescence and mixing.

Farokhirad et al. [91] simulated the coalescence-induced self-propelled jumping of a droplet by utilizing a multiphase lattice Boltzmann method. This method was used for two identical, static micro-droplets coalescing on a homogeneous super-hydrophobic substrate over a range of Ohnesorge numbers and density ratios. The results revealed that the effect of air density was significant on detachment of the merged droplet from the substrate at the later stage of the jumping process; the larger the air density, the larger the jumping height of the droplet. The intensity of vortical structures generated inside and around the merged droplet became weaker after droplet departure as the air inertia was decreased. Similar research on coalescence-induced self-propelled droplets can also be seen in the work of Boreyko et al. [92,93], Wang et al. [94] and Wisdom et al. [95].

Zheng et al. [96] investigated both experimentally and theoretically the relaxation of the liquid bridge after the coalescence of two sessile water–glycerol mixture droplets resting on a horizontal organic glass substrate. The liquid bridge relaxed to its equilibrium shape via two distinct approaches: damped oscillation, relaxation and underdamped relaxation. If the viscosity was low, damped oscillation occurred. By using this approach, the liquid bridge underwent a damped oscillation process before it reached a stable shape. However, if the viscous effects became significant, underdamped relaxation occurred. Their further analysis indicated that the damping rate and oscillation period of damped oscillation were related to an inertial-capillary time scale *τ_c_*. Chireux et al. [97] also did similar research, in which the inertial oscillations of a liquid bridge maintained between two droplets under the condition of negligible gravity were studied both experimentally and theoretically.

Due to the various surface properties of solid substrates, there are different values of contact angle and contact angle hysteresis for the sessile droplets resting on them. These droplets can be simply forced to coalesce or merge through condensation growth and syringe injection. The contact line between the droplets and the solid substrate greatly slows down the coalescence process and the relaxation of the merged droplet. At early stages of the droplet coalescence, a traveling wave propagates from the initial contact point of two droplets as the core liquid is drawn into the bridge region. When this traveling wave reaches the apex of each side, the heights of the droplet start to decrease. The growth rate of the meniscus bridge not only heavily relies on the droplet radii, but also the heights of the droplets at contact. There are two distinct approaches for relaxation of the liquid bridge: damped oscillation relaxation for inertial coalescence and underdamped relaxation for viscous coalescence.

#### 2.1.3. Coalescence of a Falling Droplet and a Sessile Droplet on a Solid Substrate

Apart from the research on the coalescence of two sessile droplets that approach each other via condensation growth or syringe injection, there have also been a certain number of studies focusing on a falling drop colliding with a sessile drop on a solid substrate. Such coalescence is easy to implement under the effect of gravity and gives rise to practical applications of droplet-based micromixing and microreaction.

**Figure 12:**
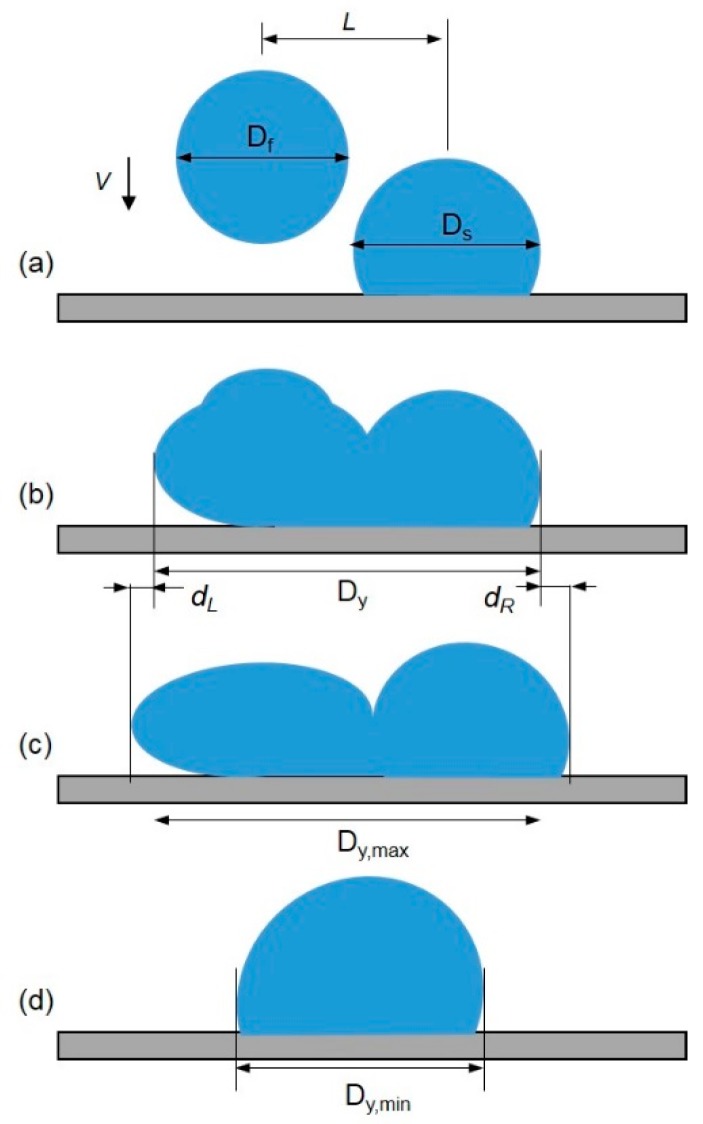
Sketch of (**a**) the deposition of two droplets on a solid surface; *D_f_* is the diameter of the falling droplet; *D_s_* is the diameter of the sessile droplet; and *L* is the center-to-center distance; (**b**) Spread length *D_y_*; (**c**) maximum spread length *D_y,max_*; (**d**) minimum spread length *D_y,min_*.

**Figure 13:**
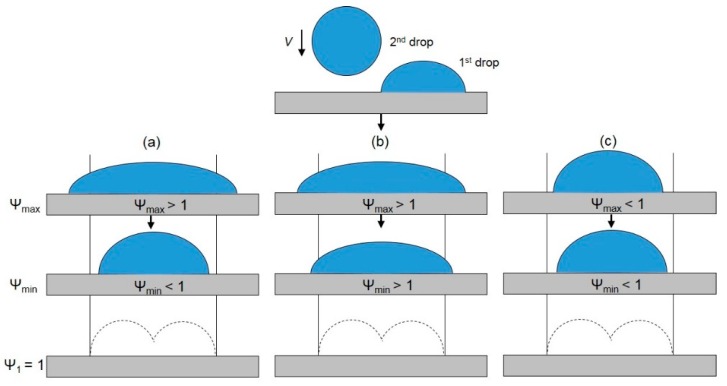
Schematic representation of coalescence mechanisms: (**a**) drawback due to retraction; (**b**) additional spread; (**c**) drawback not due to retraction.

Li et al. [38] designed a series of experiments to study the coalescence of a falling droplet with a stationary sessile droplet on a stainless steel surface under isothermal conditions (Figure 12). By analyzing high-speed images, the contact line movement was found to be related to the change of the local dynamic contact angle and the evolution of free surface. It was also found that the spread length could be either larger or smaller than the ideal spread length, which was defined as the spread diameter of an individual droplet plus or minus the center-to-center distance between the two droplets. The authors thus identified three different coalescence mechanisms based on comparing the maximum and the minimum spread lengths to the ideal spread length (Figure 13). Liang et al. [98] observed the process of a single liquid drop impinging upon a static hemispherical drop on steel spheres, in which a glycerol–water mixture and water were selected as experimental fluids. By increasing the Weber number, they observed three outcomes after collision: rebound, coalescence and the circular liquid sheet. Similar research on the impact between droplets and the solid substrate can be also seen in Roisman et al.’s work [99], in which they focused on the experimental observation and numerical modeling of the impact of multiple droplets on a dry steel substrate.

Furthermore, Farhangi and Graham et al. [100,101] investigated both experimentally and numerically the coalescence of a falling droplet with a sessile droplet on solid surfaces with various wettabilities. A two-phase volume of fluid method was not only used to simulate the dynamics of droplet coalescence, shape evaluation and contact line movement, but also to predict the maximum spreading length of two coalescing droplets. After the falling droplet impacted and merged with the stationary sessile droplet, the inertia of the falling droplet caused the merged drop to deform and spread. After reaching the maximum spreading, surface forces caused the droplet to recoil. At this point, the droplet height started to rise slightly before falling under the influence of gravity. This process continued until the drop reached its equilibrium state. They also studied the effects of different parameters on the maximum spreading length and found that by increasing the hydrophobicity and offset ratio, the maximum spreading length decreased, while the droplet inertia had a reverse effect on the maximum spreading. Similar work has also been done by Wakefield et al. [102], who studied the concentric impact between a falling water droplet and a sessile water droplet on a Teflon substrate. Their research primarily focused on the influence of the Weber number on droplet spreading.

**Figure 14:**
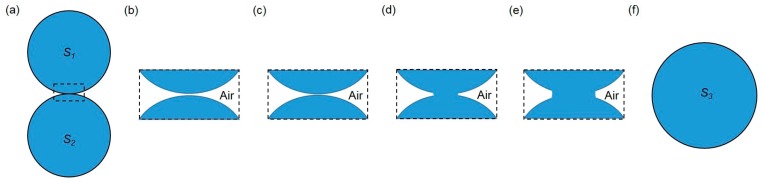
Schematic of the whole coalescence process of liquid droplets. (**a**) Two contacting liquid droplets with surface area *S*_1_ and *S*_2_ respectively; (**b**) magnification of contact area with an air layer separating two droplets; (**c**) the air layer gets thinner as the shape deformation of droplets continues; (**d**) the rupture of the interface and the appearance of a liquid bridge; (**e**) the liquid bridge grows exponentially following a certain power scaling law; (**f**) the formation of a bigger daughter droplet with a smaller total surface area (*S*_3_ < *S*_1_ + *S*_2_).

In summary, when two liquid droplets touch each other, no matter if induced by vertical collision under the effect of gravity, horizontal condensation growth or syringe injection, the droplets tend to merge. As a result, a larger daughter droplet with a smaller total surface area is formed through an initially singular motion driven by surface tension. At the initial stage of droplet coalescence, the interface between two droplets gets thinner as the shape deformation of droplets continues. Once the thickness decreases to a critical point, film drainage happens. After the rupture, a bridge (or a neck) connecting these two droplets appears and grows exponentially following a certain power scaling law. In the final stage of droplet coalescence, the merged droplet oscillates and slowly evolves to an equilibrium shape (Figure 14).

### 2.2. Mechanisms of Liquid Marble Coalescence

Compared to the extensive studies on droplet coalescence, there has been little research on LM coalescence, which has mainly focused on the impact between LMs and solid surfaces, vertical collisions of LMs, and external-field-induced coalescence of LMs.

Planchette et al. [103] investigated the properties of LMs coated by hydrophobic particles when impacting on to a solid substrate in a large range of impact velocities. The authors characterized three different behaviors during the impact, namely non-bouncing, bouncing and rupture, and also defined transition boundaries between these three regimes. At a small impact velocity, the surface energy stored during impact was not large enough to overcome gravity plus oscillation energies and there was no bouncing. At a large impact velocity, rupture of the surface coverage occurred, which prevented the droplet from integer bouncing. By comparing the impact of a LM on a smooth surface with that of a bare water droplet on a rough superhydrophobic surface, it was clearly found that the presence of particle coverage greatly improves the stability of small liquid volumes.

Supakar et al. [104] also identified the details of the impact of LMs on solid surfaces by using dual-view high-speed imaging. It was observed that particles at the surface flew rapidly to the periphery of the drop during the spreading stage. They found a power-law scaling for the normalized maximum spread and the impact Weber number, *D_max_*/*D*_0_∼*We*_α_, with *α* = 1/3. By using hydrophilic target surfaces, the marble integrity was lost even at moderate impact speeds, where the particles at the surface separated and allowed liquid–solid contact to occur. It is noteworthy, however, that there was no significant difference in the maximum spread length between hydrophobic and hydrophilic surfaces, which was rationalized by the presence of the particles.

**Figure 15:**
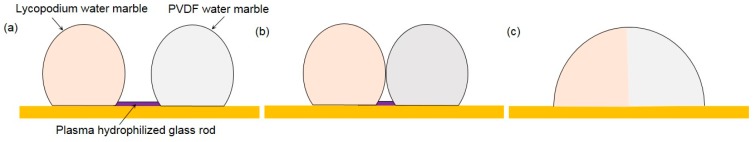
Schematic of forced coalescence of 30 μL water marbles connected with the plasma hydrophilised glass rod. The orange marble is coated with lycopodium, the silver marble is coated with polyvinylidene fluoride (PVDF). (**a**) Marbles are connected with a glass rod (depicted in purple); (**b**) water wets the rod and marbles approach one another; (**c**) the final state of the coalescence.

In terms of the coalescence of LMs, Bormashenko et al. [105] prepared water and di-iodomethane marbles enwrapped with polytetrafluoroethylene (PTFE) powder, then brought them into contact, and slightly pressed one to another. The pressed marbles coalesced, giving rise to the composite LM. They further reported forced coalescence of LMs [106]. LMs were connected by a glass rod hydrophilised with cold radiofrequency plasma, (Figure 15). The process resulted in the formation of Janus-marbles. “Sandwich” marbles enclosing solid foamed polystyrene particles and built from immiscible liquids were also reported in their study.

**Figure 16:**
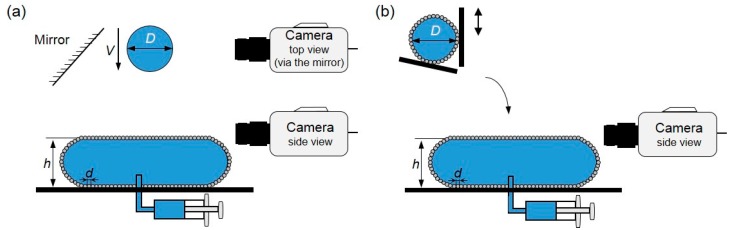
Sketch of impacts between armored interfaces recorded by a high-speed camera. (**a**) Single armor experimental set-up: a bare water droplet impacts on a water puddle armored with particles of diameter *d*. Two synchronized high-speed cameras record side and top views of the impacts; (**b**) double armor experimental set-up: a liquid marble (LM) impacts on the armored interface of another larger LM.

Planchette et al. [107] studied, respectively, the impact of a droplet and a LM on a puddle with a flat armored interface, i.e., a sessile liquid marble, to test the robustness of the interface (Figure 16). Two distinctive regimes were observed in their experiment. Small drops impacting at low velocities did not coalesce while bigger drops falling at higher velocities led to coalescence. The coalescence that occurred when the impacting drop just reached its maximum extension directly resulted from the formation of bare regions within the armor. Consequently, they proposed a geometric criterion to describe this transition, which is able to capture the dependence of the measured velocity threshold on particle sizes and droplet diameters. It is reasonable to predict that the additional robustness experienced by double armors (marble-to-marble) results in an increase of the measured velocity threshold. However, the horizontal velocity induced by a non-vertical collision was not taken into account in this experiment.

Liu et al. [108] concentrated on the coalescence of liquid water marbles driven by a DC electric field. They found that two contacting LMs could be forced to coalesce when they were charged by a sufficiently high voltage. The threshold voltage leading to the electro-coalescence sensitively depended on the stabilizing particles as well as the surface tension of the aqueous phase. By evaluating the electric stress and surface tension effects, they attributed such coalescence to the formation of a connecting bridge driven by the electric stress. This liquid bridge subsequently grew and led to the merging of the marbles. In addition, it was found that multiple marbles in a chain could be driven to coalesce by a sufficiently high threshold voltage that increases linearly with the number of the marbles. The authors further proposed a simple model to predict the relationship between the threshold voltage and the number of LMs, which is highly consistent with their experimental results.

In conclusion, the coalescence process of LMs is similar to that of droplets, but much harder to achieve due to the presence of protective particle surface coverage. When two LMs collide with a certain initial velocity, the particles at the surface flow rapidly to the periphery of the marbles due to the fluid flow inside the marbles or external forces, and bare regions appear on the interface that lead to the occurrence of liquid–liquid contact. After this initial contact, the rest of the coalescence process is almost the same as that of droplet coalescence. It is hoped that the stability of LMs could be utilized to perform different biological assays in a harsh environment.

### 2.3. Experimental Parameters to Be Considered in Coalescence Processes of Droplets and Liquid Marbles

The experimental conditions of droplet coalescence should be controlled accurately to ensure a smooth experiment on related dynamics and mechanisms. Thus, there are many experimental parameters to be considered during the coalescence process, such as different droplet sizes, impact velocities, off-center distances, properties of drop fluids (droplet viscosity and surface tension) and resting surfaces (contact angle and contact angle hysteresis), and ambient environments [34,65,90,94,109,110,111,112,113]. In addition, there are two more parameters that should be noticed in LM coalescence, namely the varieties of hydrophobic powders and liquid core inside LMs.

## 3. Engineering Coalescence of Droplets and of Liquid Marbles 

In general, inducing factors of droplet coalescence can be categorized as passive and active types based on different working principles. Passive droplet coalescence techniques do not require external energy and the droplet coalescence process only relies on the structure designs [114] and surface properties [115] of microchannels. In contrast, active droplet coalescence techniques employ energy generated by an external field to induce the coalescence process. This can be achieved by applying gravitational, electric, magnetic and thermal fields, etc. However, for LM coalescence, there are only active types of techniques in practical use. Active techniques for droplet coalescence and LM coalescence will be discussed in detail below. Among these active techniques, droplet coalescence and LM coalescence induced by an electric field are the most commonly used method.

### 3.1. Electric Energy

Electro-coalescence (EC) is an active method that is widely used to realize droplet coalescence in an electric field. In particular, EC of water microdroplets in a carrier liquid in microfluidic channels is a promising technique that enables droplet-based mixing functionalities to be achieved in LOC applications. Many researchers have made great efforts to investigate EC in microfluidics in the last two decades [116,117,118,119].

Chabert et al. [120] first presented a system applicable to EC of microfluidic droplets immersed in an immiscible solvent. The electrodes used in the system were not in direct contact with the carrier liquid or the droplets, which therefore minimized the risk of cross-contamination between different coalescence events. This capillary-based system is suitable for further miniaturization to apply in any LOC application, where the conductivity of droplets is much greater than that of the stream containing them. Furthermore, Link et al. [121] demonstrated the concept of EC again by polarizing droplets through applying an DC field. Zagnoni et al. [43] investigated the EC mechanism of microdroplets using localized electric fields. The localized electric system was found to be effective in merging droplets regardless of the distance between them. In this EC process, the viscosity of the continuous phase was dominant.

By using microfluidic chips, Thiam et al. [122] investigated the stability regarding the coalescence of a pair of droplets under an electric field as a function of the droplet–droplet distance and AC field intensity. Three different regimes were found: non-coalescence, coalescence and partial merging. They thus proposed a destruction mechanism for a macroscopic emulsion, which includes the packing condition for which total and immediate destruction is effective. Furthermore, Chen et al. [117] investigated the EC of Pickering emulsion droplets. Under a sufficiently high electric field, the originally stable droplets coalesced via two distinct approaches: normal coalescence and abnormal coalescence. In the normal coalescence, a liquid bridge grew continuously and two droplets merged together. It is similar to the classical picture depicted in previous research. In the abnormal coalescence, however, the bridge failed to grow indefinitely. Instead, the bridge broke up spontaneously due to the geometric constraint from particle shells.

Although most electric-field-induced motions have been regarded as favoring droplet coalescence, Ristenpart and his co-workers [123] reported the existence of a critical field strength above which oppositely charged drops did not merge. The authors observed that appropriately positioned and oppositely charged drops migrated towards one another in an applied electric field. However, while the drops coalesced as expected at low field strengths, they were repelled from one another after contact at higher field strengths. Qualitatively, the droplets appeared to bounce off each other. Ristenpart et al. proposed that the temporary meniscus bridge between the bouncing drops was unstable with respect to capillary pressure when it formed in an electric field exceeding a critical strength.

As with EC, dielectrophoresis (DEP) is also an effective way to realize droplet coalescence. The key difference between DEP and EC is that DEP can only occur in a non-uniform applied electric field while EC can occur in both uniform and non-uniform contexts. DEP relies on the different dielectric constant between the droplet and the surrounding medium, while EC relies on different conductivities of the droplet and the continuous phase [47]. Droplet manipulation through DEP was demonstrated by Schwartz et al. [124], Singh et al. [125], and Wang et al. [126]. In a typical scenario, an electric stress will act on a droplet surface that is subjected to a non-uniform electric field. A net electric force that is referred to as DEP force will cause the droplet motion.

**Figure 17:**
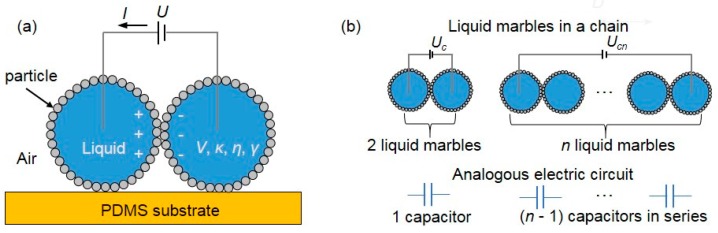
(**a**) Schematic of the coalescence of two charging LMs resting on a polydimethylsiloxane (PDMS) substrate. *V*, *κ*, *η*, *γ* are the volume, electrical conductivity, viscosity and surface tension of the liquid core respectively. The voltage *U* is applied through the electrodes inserted into the liquid marbles. The current *I* is monitored through an ammeter during the coalescence process; (**b**) sketch of a chain of liquid marbles charged by a voltage and its analogous electric circuit.

There is very limited research on LM coalescence and thus fewer papers on EC of LMs. As already mentioned above, Bormashenko et al. [105] studied the electric-field activation of composite LMs comprised of di-iodomethane and water and coated by hydrophobic powders. The authors proposed a dimensionless constant to describe the sensitivity of LMs to an electric field. Furthermore, they demonstrated the deformation of LMs in a uniform electrical field [127]. Liu et al. [108] found that two contacting LMs could be forced to coalesce when they were charged with a sufficiently high voltage, which sensitively depended on the stabilizing particles as well as the surface tension of the aqueous phase (Figure 17a). By evaluating the electric stress and surface tension effect, they attributed such coalescence to the formation of a connecting bridge driven by the electric stress. This liquid bridge subsequently grew and led to the merging of the marbles. In addition, multiple marbles in a chain could be driven to coalesce by a sufficiently high threshold voltage that increases linearly with the number of the marbles, (Figure 17b). The concept of EC of LMs could be useful in microreactors for chemical and biomedical reactions involving multiple reagents.

### 3.2. Thermal Energy

Beside electrically induced droplet coalescence, thermally induced droplet coalescence is also an effective method. The mechanism of this method is to exploit temperature-dependent viscosity and surface tension. On the one hand, increasing temperature leads to a decrease in viscosity of the continuous phase. As a result, the continuous phase flows faster. On the other hand, the surface tension of the droplet will decrease as temperature increases. The droplet coalescence process can thus take place more easily [47].

**Figure 18:**
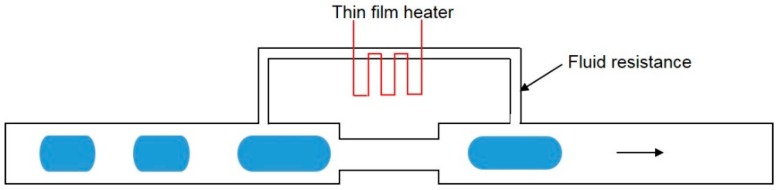
Schematic illustration of thermally controlled droplet coalescence microfluidic device.

Thermally-actuated droplet coalescence was demonstrated by Köhler et al. [128]. A high fluid resistance element was incorporated with a long channel with reduced cross section. A thin film heater was embedded within the high fluid resistance element (Figure 18). As the fluid flowed over this channel, a portion of the continuous phase would go through the small channel with high fluid resistance. As the thin film heater was activated, the temperature in the small channel with high fluid resistance increased. Meanwhile, the viscosity of continuous phase would decrease with more continuous phases going through the small channel. With the drainage of the continuous phase in the main channel, subsequent droplets would have a chance to contact each other. After droplet coalescence occurred, the heater was turned off to let the fused droplet pass through the channel.

### 3.3. Pneumatic Energy

**Figure 19:**
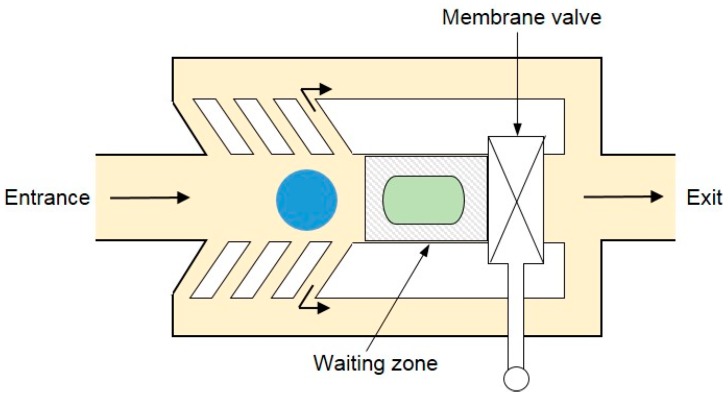
Schematic illustration of the pneumatically actuated droplet coalescence.

Another method for the active merging of droplets is pneumatically actuated droplet coalescence. In such a process, a pneumatically actuated membrane valve was constructed on the top of the specially designed microfluidic merging chamber [129]. As the droplet came into the merging chamber, the membrane valve was activated and impeded the droplet motion. The droplet then slowed down and waited for the subsequent droplet in the waiting zone. The subsequent droplet driven by the continuous phase would continue to push the former droplet moving further into the waiting zone. As they approached each other, the droplet coalescence took places. As the desired droplet coalescence was achieved, the pneumatically actuated membrane valve would open to let the fused droplet get out (Figure 19).

### 3.4. Magnetic Energy

**Figure 20:**
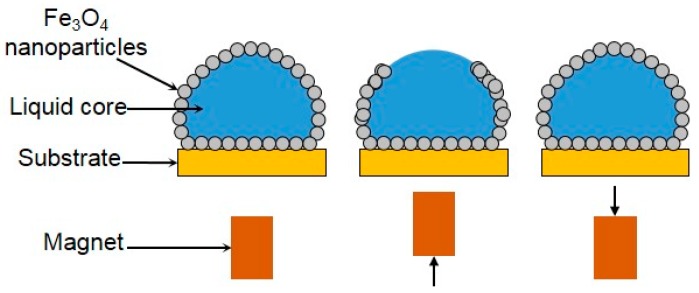
Sketch of magnetic manipulation of LMs coated with Fe_3_O_4_ nanoparticles. From left to right, the Fe_3_O_4_ nanoparticles are concentrated downwards when a magnet is brought into proximity. The process is reversible as the nanoparticles reassembled themselves after the magnet is removed.

There is also an important method for active LM coalescence through magnetic actuation. Lin’s research team [60,130,131] focused their study on the magnetic LMs coated with magnetic particles or consisting of magnetic liquid, which could work as a microreactors. These LMs could be manipulated and controlled to open and close reversibly in a magnetic field, which greatly facilitates the addition of reagents and the removal of products (Figure 20). Therefore, when two LMs were controlled to open to each other, leading to liquid core contact, the controlled coalescence of LMs was easy to achieve.

### 3.5. Optical Energy

Kavokine et al. [59] demonstrated the light-driven transport of floating LMs, which could be used for actuating LM coalescence. In their experiment, LMs were deposited on a water solution containing photosensitive surfactants. It was found that irradiation of the solution generated photoreversible Marangoni flows that transported LMs toward UV light and away from blue light when the liquid substrate was thick enough (Marangoni regime). Below a critical thickness, LMs moved in an opposite direction to that of the surface flow at a speed increasing with decreasing liquid thickness (anti-Marangoni), which was driven by the free surface deformation.

### 3.6. Other Energies

The presence of surfactants in microfluidic devices is beneficial for promoting the stability of droplets. Therefore, adding chemical reagents to react with surfactants can also induce droplet coalescence in microchannels. Besides, Sartori et al. [132] reported that the droplet motion on an inclined substrate induced by vertical vibrations could be used to initiate droplet coalescence. Aussillous and Quere [51] mentioned the actuation of LMs by gravity, which implied the application of the gravitational field in LM coalescence.

## 4. Practical Applications of Droplet Coalescence and Liquid Marble Coalescence

In addition to the various applications of droplet coalescence as mentioned in Section 1, this manipulation process also plays an important role in DMF. In particular, droplet coalescence enables desired microfluidic functions such as micromixing and microreaction to be implemented in LOC devices for chemical and biological applications. For instance, Guo et al. [22] argued that droplets allow sample volumes to be significantly reduced for biological analysis, allowing for high-throughput screening and sensitive assays based on droplet coalescence. According to their study, the manipulation and measurement of droplets at kilohertz speeds enabled up to 10^8^ samples to be screened in one day. Mashaghi et al. [2] reported that droplet-based laboratory operations on a small scale provided numerous benefits such as reduced quantities of reagents and waste and increasing portability and controllability of the assays. These operations often involved mixing and reaction of discrete droplets, which require the coalescence process. The authors highlighted that there will be more applications of droplet microfluidics in chip-based technologies such as single-cell analysis tools, cell cultures, in-droplet chemical synthesis, high-throughput drug screening and nanodevice fabrication.

**Figure 21:**
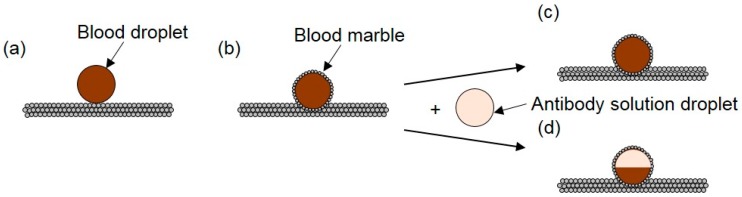
Schematic illustration of the microbioreactor for blood-type identification. (**a**) A blood droplet (10 μL) is placed on a hydrophobic precipitated calcium carbonate powder bed to form the corresponding blood marble; (**b**) a droplet of antibody solution (10 μL) is then injected inside the blood marble to complete the preparation of the microbioreactor; (**c**) when the corresponding antigens are not present on the surface of red blood cells, no separation is visible; (**d**) when the corresponding antigens are present, red blood-cell agglutination reaction will take place, which will result in the separation of marble color into two distinct light (top) and dark (bottom) parts.

Due to its unique properties, the LM as an emerging digital microfluidic platform has attracted extensive attention from researchers in the past decade, mainly in fundamental research with some practical uses such as the transport of a small liquid volume without any leakage, detection of water pollution, gas sensing, gas–liquid reactions and microreactions. As one of the most important microfluidic components, a microreactor is the platform that significantly benefits from LM coalescence. Each LM functions as a miniature chamber containing different reagents. Using external-field-induced coalescence, LMs can merge to form a bigger daughter LM so the reagents can mix and react. LM coalescence also allows the daughter LM to have all the related properties of the parent LMs. Arbatan et al. [133] selected human blood grouping (ABO and Rh) as the biological system to demonstrate the use of LM as a microbioreactor in practical diagnosis involving human blood (Figure 21). They summarized several significant advantages of a LM-based microbioreactor as following: First, it requires relatively small amounts of samples and reagents. Second, it reduces biohazards, since the powder-enwrapped biological sample does not contact with the supporting substrate. Third, the control of bioreactions can be made by either coalescing marbles containing different reagents or by injecting different reagents into one marble. Fourth, marbles cost less. They further exploited the possibility that LMs worked as efficient miniature bioreactors inoculated with Hep G2 cells to culture cell spheroids in vitro and found this idea works well.

Tian et al. [134] demonstrated the possibility of using LMs to build respirable micro-biological reactors to cultivate microorganisms, which was attributed to the porous and superhydrophobic shell of LMs that prevented the liquid core inside from contacting surfaces outside, but allowed gases to transport freely across the shell. Recently, Vadivelu et al. [135,136] have demonstrated the use of LMs as a three-dimensional cell culture platform for various cells such as olfactory ensheathing cells, which mainly relied on LM coalescence. In addition, Lin’s research team [60,137] investigated the optical detection of magnetic LMs for possible application as a new discrete microfluidic system, which might form a new platform technology for using the magnetic LM as a “smart” microreactor for chemical analysis or exploring new chemical reactions, especially when multiple precious, explosive or highly toxic reagents are used and the products need to be stored without separation.

Beside several applications mentioned above, LMs can be also used for the fabrication of color pigments [138] and drug discovery utilizing hollow granules formed by LMs’ evaporation [139]. Some systematic review papers on the practical applications of LMs in chemical, biological and pharmaceutical assays have been published recently [135,140,141].

## 5. Conclusions and Future Perspectives

Droplet coalescence has been systematically studied in the last few decades due to its extensive applications in industries, particularly in chemical and biological analysis. It can be categorized into coalescence of two droplets freely suspended in various fluids; coalescence of two sessile droplets on a solid substrate via either condensation growth or syringe injection; and coalescence of a falling droplet and a sessile droplet resting on a solid substrate. There are many experimental parameters to be considered during coalescence, which include droplet sizes, impact velocities, off-center distances, the properties of droplet fluids and resting surfaces, and ambient environments. External fields, such as the electric field and the thermal field, have been adopted to induce droplet coalescence. Drawing from the understanding of droplet coalescence, LM coalescence has been further studied due to the recent promising development of LMs. Based on the few studies on LM coalescence, it is clear that the coalescence process of LMs has a number of similarities with the coalescence process of droplets, but is much harder to achieve, which is attributed to the presence of the protective particle layer. Research on external-field-induced coalescence of LMs is limited, mainly focusing on EC and magneto-coalescence. As a novel digital microfluidic platform, the LM has broad practical applications ranging from, but not limited to, the displacement of a small volume of liquid without any leakage, detection of water pollution, gas sensing, gas–liquid reactions and more importantly, microreactions.

Although research on LMs has a short history, their unique properties have made the LM an active objective in chemical and biological analysis. However, there is little systematic research on LM coalescence. For future study, there are several viable research options worth exploring. When LMs are compressed upon each other, much less is known about the motion and distribution of particles on the interface, where the ultra-high-speed imaging and other fast detection methods are needed. In terms of LM coalescence by vertical collisions, how to transport the LM from one point to another precisely without serious damage is still an open question. Nowadays, electric-field-induced coalescence of LMs relies on DC power. Whether an AC electric field can be used to induce LM coalescence needs to be further investigated. In addition to the coalescence of LMs, mixing inside LMs is also of great importance in biological applications. External-field-induced mixing inside LMs, such as the electric field and the magnetic field, could be the focus of future research.
